# Resistance Evolution to *Bt* Crops: Predispersal Mating of European Corn Borers

**DOI:** 10.1371/journal.pbio.0040181

**Published:** 2006-05-30

**Authors:** Ambroise Dalecky, Sergine Ponsard, Richard I Bailey, Céline Pélissier, Denis Bourguet

**Affiliations:** **1**Institut National de la Recherche Agronomique (INRA), UMR Centre de Biologie et de Gestion des Populations (CBGP), Campus International de Baillarguet, Montferrier-sur-Lez, France; **2**Laboratoire Dynamique de la Biodiversité, CNRS-Université P. Sabatier–Toulouse III, Toulouse, France; University of TennesseeUnited States of America

## Abstract

Over the past decade, the high-dose refuge (HDR) strategy, aimed at delaying the evolution of pest resistance to *Bacillus thuringiensis (Bt)* toxins produced by transgenic crops, became mandatory in the United States and is being discussed for Europe. However, precopulatory dispersal and the mating rate between resident and immigrant individuals, two features influencing the efficiency of this strategy, have seldom been quantified in pests targeted by these toxins. We combined mark-recapture and biogeochemical marking over three breeding seasons to quantify these features directly in natural populations of *Ostrinia nubilalis,* a major lepidopteran corn pest. At the local scale, resident females mated regardless of males having dispersed beforehand or not, as assumed in the HDR strategy. Accordingly, 0–67% of resident females mating before dispersal did so with resident males, this percentage depending on the local proportion of resident males (0% to 67.2%). However, resident males rarely mated with immigrant females (which mostly arrived mated), the fraction of females mating before dispersal was variable and sometimes substantial (4.8% to 56.8%), and there was no evidence for male premating dispersal being higher. Hence, O. nubilalis probably mates at a more restricted spatial scale than previously assumed, a feature that may decrease the efficiency of the HDR strategy under certain circumstances, depending for example on crop rotation practices.

## Abstract 2

Mark-recapture experiments reveal that the European corn borer mates locally in their natal cornfield, which may limit the efficacy of management strategies to delay resistance to *Bt* insecticidal toxins produced by transgenic crops.

## Introduction

Right after emergence and just before mating, adult insects face a crucial dilemma that The Clash [ 1] celebrated in a famous song that could be slightly rephrased as: “Should I mate or should I go now? If I mate there will be trouble. But if I go it might be double.” In other words, fitness is affected by two important adult “decisions”—mating and dispersal—and by the order in which they are performed. Mating before dispersal may increase the risk of consanguinity, but dispersal before mating may increase the risk of not finding a sexual partner. Therefore, the timing between mating and dispersal is likely to vary with species and environmental conditions [ 2]: in the absence of a universal optimal strategy, no general prediction can be made and each species of interest must be studied on its own.

Nevertheless, the timing between mating and dispersal can be of great practical importance: notably, it may influence the efficacy of strategies intended to drive the microevolution of agricultural pest species—e.g., the evolution of their resistance to control agents such as pesticides—by managing agricultural landscapes [ 3]. The “high-dose refuge” (HDR) strategy [ 4] is one such strategy. It is aimed at delaying or preventing the evolution of resistance in target pest populations against *Bacillus thuringiensis (Bt)* toxins produced by transgenic *Bt* crops [ 5]. The underlying principle is that, in a patchy environment of treated and untreated areas, high gene flow between patches with different selection pressure (here, between *Bt* crop fields and *Bt*-free refuges) should limit local adaptation [ 6] (here, the selection of resistance alleles). Over the past decade, an HDR-based management of agricultural land became mandatory for several *Bt* crops in the United States [ 5] and is being discussed for Europe [ 7].

Population genetics studies conducted on the main pests targeted by the *Bt* crops usually revealed no departure from patterns expected at Hardy-Weinberg equilibrium and suggested a high level of gene flow over a broader spatial scale than that of the patches, a necessary condition for the success of the HDR strategy (e.g., [ 8– 10]). However, tests for departure from Hardy-Weinberg equilibrium are quite conservative, estimates of the spatial scale at which gene flow occurs based on such methods are coarse, and, more fundamentally, the same genetic structure patterns can also be generated by a number of alternative processes [ 11].

In particular, population genetics studies provide no information on whether adults mate mostly before or mostly after dispersal. However, high predispersal mating can reduce the efficacy of the HDR strategy. Indeed, the goal of this strategy is to purge each generation of as many as possible of its *“r”* alleles (alleles conferring resistance to the *Bt* toxin) to counterbalance, as much as possible, the increase of their frequency resulting from the selection pressure exerted by *Bt* crops. The *r* alleles are carried by either heterozygous *(rS)* or homozyous *(rr)* individuals. Resistance to *Bt* toxins being generally recessive [ 12], only the fraction of *r* alleles carried by *rS* individuals among the offspring of any given generation is thus available for purging. Adults emerging from a *Bt* field are likely to be mostly resistant homozygotes *(rr),* whereas adults emerging from refuges are mostly susceptible homozygotes *(SS).* If most mating takes place before dispersal, the proportion of heterozygotes in the offspring is expected to be low. Other things being equal (but see, e.g., [ 13] and Discussion), the higher the predispersal mating rate, the lower is the expected *rS*/( *rS* + *rr*) ratio in the offspring and the lower is the expected success of the HDR strategy.

Classical mark-recapture studies have been performed on a number of agricultural pests (e.g., [ 14– 16]), mostly with the aim of estimating dispersal distances. Although they provide more precise estimates of such distances than population genetics studies, only very few of them examined the timing between mating and dispersal. This relationship has, to our knowledge, seldom and incompletely been quantified directly in natural populations of insect pests targeted by *Bt* crops (but see [ 16– 18]). First, it is often difficult to detect individuals in the field at the very moment they mate, and even more difficult to appreciate their mating success in terms of sperm transfer. Second, and most important, the origin—local or external—of individuals mating in a specific site can generally not be ascertained.

The present study deals with the European corn borer (ECB), Ostrinia nubilalis Hübner (Lepidoptera: Crambidae), one of the major pests of corn ( Zea mays L.), and one of the main targets of the Cryab and Cry1F *Bt* toxins produced by transgenic *Bt* corn [ 19]. Since the 2000 growing season, US corn belt growers planting *Bt* corn outside and inside *Bt* cotton growing areas must assign 20% and 50%, respectively, of their corn acreage to refuges, and must set these refuges less than 0.5 mile (approximately 800 m)—and preferably less than 0.25 mile (approximately 400 m)—away from *Bt* cornfields [ 20]. When these requirements were made, empirical data about ECB dispersal were very scarce [ 20, 21]. Studies published since then [ 22– 24] confirmed that dispersing ECB moths are able to move over far more than a few hundred meters, but the proportion of individuals actually engaging in dispersal, the probability that they do so before versus after mating, and the probability that immigrants and residents mate randomly remained open questions.

We used a combination of color-mark, release and recapture experiments and of biogeochemical marking [ 25] over three breeding seasons (one in 2004 and two in 2005) to quantify, directly in natural populations, the percentage of ECB individuals staying long enough to mate in their natal cornfield and its herbaceous border and the percentage of mating actually occurring between those individuals when mixed with dispersing individuals coming in from surrounding cornfields. Our results show that immigrant males, once present locally, have the same probability of mating with resident females as resident males have, while limited evidence suggests that immigrant females mate less readily with local males than do resident females. They also show that a variable and sometimes substantial proportion of ECB females mate before they engage in any dispersal, with no evidence for any sex-related difference. This might decrease the efficiency of the HDR strategy in certain situations.

## Results

### Experiment 1

Experiment 1 aimed at estimating the probability of young (less than 24 h old) individuals located in a cornfield to be found in the closest field border two nights later and at estimating the probability that such young individuals mate assortatively rather than with immigrants coming from other cornfields. Over 17 sessions spread over 3 wk of the June 2004 breeding season, we released a total of 8,788 virgin moths into four different cornfields ( Table 1). These fields were planted with soybean ( Glycine max L.) or wheat ( Triticum sativum L.) during the previous (2003) season (two plants that suffer no or very little infestation by the ECB [ 26, 27]), thus ensuring that virtually no ECB pupae from the previous season were present locally. In this respect, such fields mimicked fields planted with *Bt* corn during the previous season. The closest fields planted with corn in 2003—mimicking the refuges—were located at a distance of at least 400 m. ECB larvae having overwintered in these 2003 cornfields were the closest, and hence the most likely, sources of wild moths occurring at the study sites in 2004.

**Table 1:**
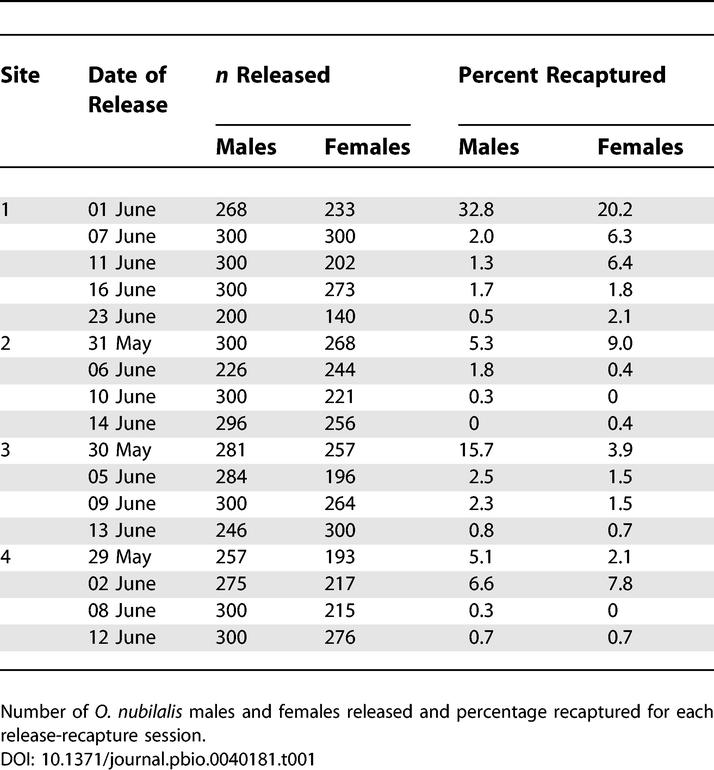
Experiment 1

At each site and for each release session, 340 to 600 marked moths that had emerged less than 24 h earlier were released into the cornfield, between the seventh and eighth rows, planted parallel to the field border ( Table 1). Released moths were marked twice: they were reared as larvae on a wheat (a C _3_ plant) diet, which caused their tissues to contain a lower ratio of ^13^C/ ^12^C carbon isotopes than wild individuals fed on corn (a C _4_ plant) [ 27– 31] without altering their longevity ( Table S1) or propensity to mate ( Table S2), and they were marked with a colored ink spot on the dorsal thorax. Thirty-six hours (2 nights and 1 day) after release, we recaptured a total of 374 marked moths in the herbaceous borders of the field, along with a total of 5,504 unmarked individuals ( Table 2).

**Table 2:**
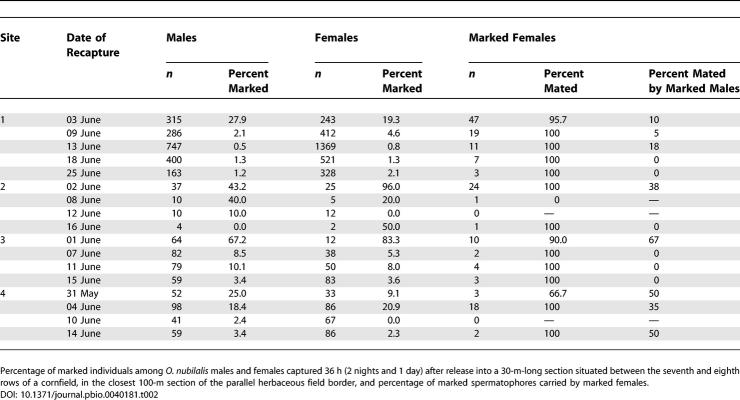
Experiment 1

The proportion of marked individuals among all moths caught varied widely between sites and sessions, as did the proportion of individuals released during a given session that were recaptured ( Tables 1 and 2). The percentage recaptured averaged (±1 SE) 4.3 ± 1.6% and ranged from 0.2% to 26.9%. This proportion was not significantly different between sexes (paired *t*-test: *t* = 0.73, *df* = 16, *p* = 0.762). Approximately 97% of the 155 females we recaptured were mated ( Table 2). To determine whether they had mated with wild or with released males, the spermatophore (or the most recent spermatophore, in cases of multiple matings) dissected from the bursa copulatrix of their genital duct were subjected to stable carbon isotope analysis [ 27, 31]. As expected, the δ ^13^C values of the 150 spermatophores showed a bimodal distribution. Based on previous results [ 27], spermatophores with δ ^13^C values of −31‰ to −22‰ were assigned to released males reared on wheat, whereas those with δ ^13^C values of −20‰ to −9‰ were assigned to wild (corn-fed) males originating from surrounding cornfields. A small number of spermatophores ( *n* = 5, less than 4%) with intermediate δ ^13^C values (−22‰ to −20‰) were considered of uncertain origin and excluded from our analysis. This biogeochemical method therefore resulted in the assignment of greater than 96% of the mated females' sexual partners to either the wild or the released males' pool.

**Figure 1:**
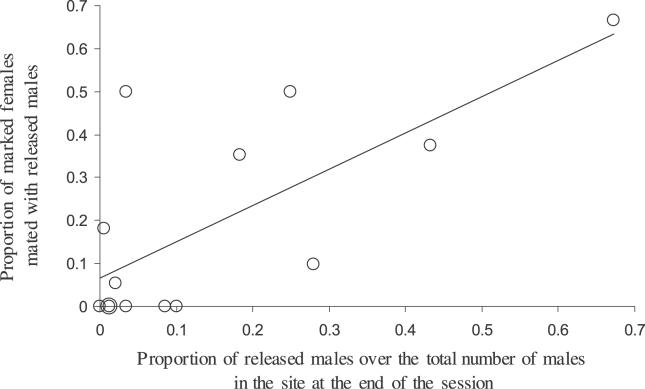
Frequency of Crossing among Resident Moths as a Function of the Local Abundance of Resident Males Experiment 1. Correlation between the proportion of marked among spermatophores dissected from marked O. nubilalis females recaptured during a given session and the proportion of marked males among the total number of males captured during recapture during the same session ( *r*_S_ = 0.622, *p* < 0.05, *n* = 14).

**Figure 2:**
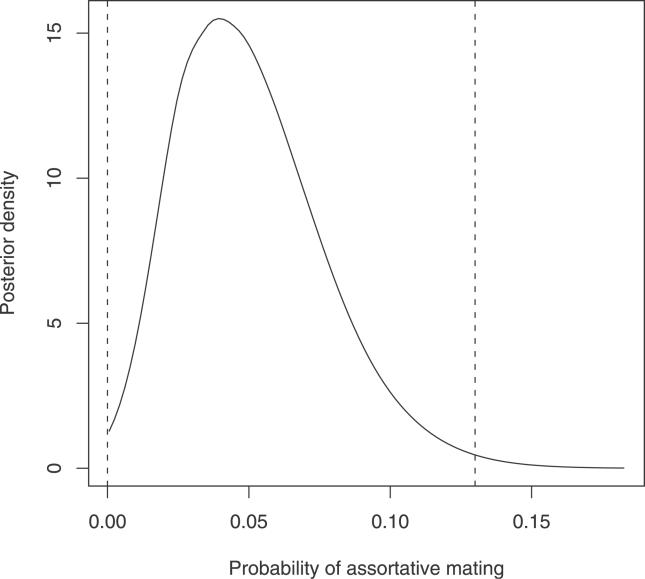
Posterior Density of the Probability of Assortative Mating Experiment 1. Posterior distribution of the probability of assortative mating ( *p _Aij_*) among released O. nubilalis moths, as estimated using a Bayesian Markov chain Monte Carlo approach (see text for details). The dashed lines indicate the 95% credibility interval (0.00 to 0.13).

Marked and recaptured females had mated with both types of males. Over all sites, the proportion of released males among their partners was 19.4%. It ranged from 0% to 67%, differing considerably over the 17 release-recapture sessions ( Table 2), and it was positively correlated with the ratio of the number of released males over the total number of males captured in the herbaceous border during the corresponding session (Spearman's rank correlation coefficient *r*_S_ = 0.622, *p* < 0.05, *n* = 14; Table 2 and Figure 1). In accordance with this result, the maximum-likelihood estimate of the proportion of released females mating assortatively with released rather than wild males was very low (mode = 0.04), and its 95% credibility interval included 0 ( Figure 2).

We also determined the δ ^13^C values of the spermatophores dissected from the bursa copulatrix of 79 wild females caught during sessions displaying the highest released/wild male ratios. We found that all but three wild females had mated with wild males and that the overall proportion of females mated with wild males was thereby significantly higher among wild females (96.2%) than among recaptured females (80.6%; Fisher's exact test, *p* = 0.0003). Wild females also carried more numerous (Fisher's global test, χ ^2^ = 19.13, *df* = 8, *p* = 0.014) and older (Fisher's global test, χ ^2^ = 71.24, *df* = 8, *p* < 0.0001) spermatophores than recaptured females did ( Tables S3 and S4). Furthermore, wild females captured in the study sites carried more numerous (Fisher's global test, χ ^2^ = 16.15, *df* = 8, *p* = 0.040) and older (Fisher's global test, χ ^2^ = 72.00, *df* = 8, *p* < 0.0001) spermatophores than did wild females caught in the border of nearby cornfields planted with corn the previous year; i.e., the closest and therefore the most likely sources of most wild moths found in the study sites ( Tables S3 and S4).

Although very informative, Experiment 1 had two drawbacks. First, although the moths we released were very young (less than 24 h postemergence) and although the proportion of recaptured moths did not vary with age upon release (less than 12 h or 12 to 24 h; see Materials and Methods), we could not entirely discard the possibility that, in natural conditions, some dispersal occurs during the very first hours of adult life and that the moths we released had already lost this propensity to disperse. Second, Experiment 1 most probably underestimated the proportion of moths mating in the vicinity of their natal field. Indeed, the herbaceous border section screened during any recapture session was only a small part of the entire field border. Moreover, given that both male and female ECBs can mate a few hours after emergence [ 32], some released moths might have mated locally and left the site before the recapture session. Experiments 2 and 3, performed the following year, were designed to fill these two gaps.

### Experiment 2

Experiment 2 was designed to characterize moth movement right after emergence, directly in field conditions. In June 2005, we monitored the time of emergence of a batch of ECB pupae placed directly in the field. A total of 221 emergences were recorded. There were two emergence peaks: 51.6% of pupae emerged in the morning (07:00 a.m. to 02:00 p.m., mostly [more than 90%] between 09:00 a.m. and 11:00 a.m.), and 48.4% emerged in the evening (04:00 p.m. to 07:00 a.m., mostly [more than 90%] between 07:00 p.m. and 03:00 a.m.).

However, regardless of their sex and period of emergence, more than two thirds of the moths performed their first flight between 09:00 a.m. and 02:00 p.m. during the first 24 h of their adult life. The time of day of the first flight did not vary with period of emergence ( *F*_1, 165_ = 0.452, *p* = 0.502), and varied only slightly with sex (females: 02:04 p.m. ± 27 min, range: 08:55 a.m. to 10:45 p.m., *n* = 68; males: 00:48 p.m. ± 17 min, range: 08:50 a.m. to 10:24 p.m., *n* = 101; *F*_1,165_ = 4.347, *p* = 0.039), with no significant interaction between sex and period of emergence ( *F*_1,165_ = 0.595, *p* = 0.442). This confirms quantitatively our observations that individuals emerging in the evening typically move relatively little during their first night, and subsequently move essentially like individuals emerging the next morning.

The average distance covered during the first flight after emergence also did not vary with period of emergence ( *F*_1,120_ = 2.309, *p* = 0.131) or sex ( *F*_1,120_ = 0.870, *p* = 0.353; effect of the interaction between sex and period of emergence: *F*_1,120_ = 0.017, *p* = 0.896). Over all moths, the average distance (±1 SE) covered during this first flight was short (0.95 ± 0.13 m, range: 0.10 to 10.20 m, *n* = 124) and not significantly different from that of subsequent daytime movements (0.94 ± 0.25 m, range: 0.10 to 10.00 m, *n* = 52; *t* = −0.034, *df* = 174, *p* = 0.973). The average time lag (±1 SE) between successive flights was 26.2 ± 1.1 min, with a 95% confidence interval of 19.3 to 40.5 min. The frequency of these flights, recorded during 136 observation sessions of 10 min each, did not vary with period of emergence ( *F*_1,123_ = 0.278, *p* = 0.599) or sex ( *F*_1,123_ = 1.067, *p* = 0.304; effect of the interaction between sex and period of emergence: *F*_1,123_ = 0.264, *p* = 0.608). Hence, even a moth performing its first flight earlier than most individuals (say, at 06:00 a.m.) and moving always in the same direction would have a 95% probability of being located less than 67 m from its emergence point at 10:00 p.m. (average ± 1 SE: 34.5 ± 16.4 m, 95% confidence interval: 3.1 to 66.9 m).

### Experiment 3

Experiment 3 aimed at examining the proportion of virgin ECB females mating locally, i.e., within 50 m of the cornstalk on which they are settled at dusk, over one night. As shown in Experiment 2, this stalk is situated less than 67 m from the moth's place of emergence in 95% cases (34.5 m on average). To this effect, we released a total of 747 less-than-24-h-old virgin females by settling them on individual cornstalks, during both the first (June) and the second (August) 2005 breeding season. In June, the virgin ECBs were obtained from an outbred laboratory strain and color-marked before release. In August, the virgin ECBs were obtained as offspring of wild moths collected during the first flight, and they were not color-marked before release, for us to be able to exclude any effect of ink or laboratory selection on our results.

During the first flight season, the average (± 1 SE) proportion of females recaptured within the release section was 36.7 ± 8.6% (range: 16.7% to 62.2%; *n* = 5 release sessions; Table 3). During the second flight, this proportion was 15.6 ± 2.5% (range: 7.6% to 27.8%; *n* = 8 release sessions; Table 3). Both proportions were significantly different from each other (Mann-Whitney test, *p* = 0.040) ( Table 3). This difference may result partly from moths being more easily overlooked in August (corn height: approximately 2.5 m) than in June (corn height: approximately 0.5 m), and partly from variations in the propensity to disperse due to environmental factors such as temperature.

**Table 3:**
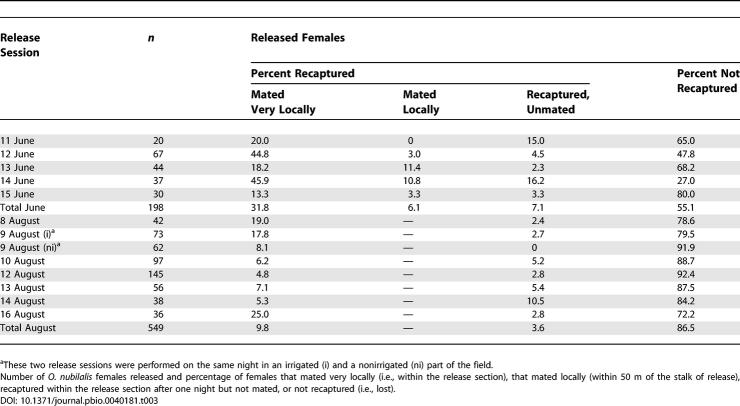
Experiment 3

During the first flight season, taking into account only females recaptured within the release section itself—often on the very stalk where they had been released—the proportion of mated females over the total number of released females averaged (±1 SE) 28.4 ± 7.0% (range: 13.3% to 45.9%; *n* = 5 release sessions; Table 3). If we include females recaptured within 50 m from the stalk of release, this estimate rises to an average of 34.1 ± 7.8% (range: 16.7% to 56.8%; *n* = 5 release sessions; Table 3). During the second flight season, the proportion of females observed mating within the release section itself averaged (±1 SE) 11.7 ± 2.7% (range: 4.8% to 25.0%; *n* = 8 release sessions; Table 3). Over both breeding seasons, the average proportion (±1 SE) of females mating locally was 18.1 ± 3.8% ( *n* = 13 release sessions; Table 3).

Experiment 3 was also attempted on virgin less-than-24-h-old males. Unfortunately, they proved to be too agitated to be settled successfully on a cornstalk at dusk. They were usually no longer present within 50 m of their initial location after approximately 30 min.

## Discussion

Since the HDR strategy has been chosen in the United States with the aim of delaying pest adaptation to transgenic insecticidal crops, a number of its potential limitations have been examined (see [ 5, 7, 33]). One of its main technical problems is that significant preferential mating among resistant individuals could decrease its efficiency [ 4]. However, despite a large modeling effort [ 4, 13, 34– 41] and despite calls for data on how far adults move before they mate in order to determine the most suitable management strategy (e.g., [ 33]), empirical studies addressing this question remained scarce.

In the case of the ECB, a major target of the *Bt* toxins produced by transgenic insecticidal corn, the few studies on dispersal that have been published so far [ 22– 24] provide estimates on the distance moved by this pest but little indication on the level of mating between immigrant and resident individuals or on the timing between dispersal and mating. These features are important because they directly influence the probability of mating between susceptible and resistant ECB moths and as such may have a significant influence on the efficiency of the HDR strategy. From that perspective, the present study generated two important findings. First, there was no evidence that the mating success of immigrant, and presumably older, males is any different from that of resident males. Indeed, at the local scale, resident females mated regardless of males having experienced a dispersal event beforehand or not. Limited evidence suggests that, at the study sites, the mating success of immigrant females may, however, be lower than that of resident ones. Second, a fraction of both male and female ECB adults mate before they engage in any long-range dispersal.

Indeed, Experiment 1 shows that virgin females mate indiscriminately with both immigrant and resident males. Our Bayesian estimate of the proportion of assortative mating among released moths was very low, less than 4%, and its 95% credibility interval included 0, suggesting that, at the local scale, resident females mate randomly with resident and immigrant males. We must, however, caution that this may not be true for resident males, as wild immigrant females carried a significantly lower proportion of marked/total spermatophores than did released females; this could be due to factors such as older age, previous matings, or decreased energy reserves of immigrant females. The generality of this result needs to be confirmed, as this feature was examined over four sessions but for only one site. However, it is consistent with the fact that, conversely, part of the released females mated before dispersal (see below), which means that they, in turn, arrived as mated immigrants in the places to which they dispersed.

Experiment 1 also shows that on average, 4%, but up to 27%, of virgin male and female ECB moths released in a cornfield can be recaptured in the vicinity of this field after two nights, with no evidence for any sex-related difference in recapture rates. Such a delay is known to be long enough for more than 70% ECB females to be mated [ 22, 42]; and indeed approximately 97% of the females that we recaptured were mated. The proportion of the recaptured males that had mated could not be determined; at least some of them did, as marked spermatophores were recovered from both marked and unmarked females. One of the limitations of this first experiment, though, is that individuals were kept in the laboratory for up to 24 h after emergence before release. Hence, we could not exclude that substantial premating dispersal in fact occurs during a short time window situated very early in adult life but that our experimental design had prevented us from detecting it.

This point was addressed in Experiment 2 using pupae placed directly into the field. The delay between emergence and first flight proved to be relatively long—on average approximately 3.5 h and 9.5 h for moths emerged in the morning and in the evening, respectively—and time intervals between subsequent movements averaged approximately 26 min. The estimated distance covered during this first flight was similar to subsequent flights and was short, on average, less than a meter. Using these values, we calculated that, at dusk, young (less than 24 h old) moths have a 95% probability of being located less than 67 m from their emergence point. Therefore, Experiment 2 showed no evidence of any high precopulatory dispersal during the very first hours of adult life.

Experiment 2 further suggested that the 4% of moths that were recaptured in Experiment 1 was an underestimate, rather than an overestimate, of the proportion of adult moths remaining locally long enough to mate. This was confirmed by Experiment 3, raising this estimate to an average of approximately 18% for female moths (approximately 34% and 12%, in June and August, respectively). Our experiments thus show that a substantial proportion of recently emerged ECB females can and do mate at a very local scale before engaging in any long-range dispersal. This conclusion is further supported by the fact that 96.2% of wild females captured during the course of Experiment 1 had mated one or several times with wild males, while only 3.8% had mated with released males. The latter estimate is significantly less than the corresponding proportion among released females (19.5%), despite the fact that this proportion was estimated over all sessions for released females but, conservatively, only over the sessions when the proportion of released over total males was highest for wild females. This suggests that a significant part of the wild females captured during the course of this experiment may have preferentially mated at the vicinity of fields from which they emerged and thus before long-range dispersal.

Hence, although the ECB is able to move over far more than a few hundred meters at the adult stage [ 22– 24], its life history may be similar to that of, e.g., Diatraea grandiosella Dyar (Lepidoptera: Crambidae), another stalk-boring moth targeted by *Bt* corn. In the latter species, more than 91% of females are mated within 24 or 48 h of emergence, 66% of females mate the first night after emergence, and precopulatory flight of males occurs mostly within the natal field (references in [ 13]). The timing between dispersal and mating has also been studied on another stem borer, *Chilo suppressalis,* a pest targeted by *Bt* rice [ 17]. Approximately 15% of females called and mated within 3 m of the site of eclosion, and approximately 5% of males mated within 5 m of the site of eclosion. In the same vein, Alyokhin and Ferro [ 16] found that a significant proportion (approximately 50%) of the Colorado potato beetles ( *Leptinotarsa decemlineata,* which is one of the pests targeted by *Bt* potato) stayed and were seen pairing close to the place of their larval development before they left or before the end of the experiment, but, unlike in the present study, it was not ascertained that the observed pairing behaviour actually resulted in sperm transfer.

What are the implications of the present findings on resistance management? One implicit—and, to our knowledge, so far untested—assumption of most models on which the HDR strategy is based is that, once present locally, immigrant individuals have the same probability of mating with any locally present individual of the other sex, regardless of its having performed dispersal beforehand or not. One of our two main conclusions is that this assumption is valid for immigrant males but questionable for immigrant females. One must keep in mind that our study was performed on susceptible moths. *Bt* resistance alleles might be associated with fitness costs that may decrease mating success. This has been shown in another lepidopteran pest targeted by *Bt* crops, *Pectinophora gossypiella,* where susceptible males were found to mate more often than resistant males in competition for matings with virgin females [ 43]. The possible impact of *Bt* resistance on ECB life history traits can currently not be evaluated since, to our knowledge, no ECB strain resistant enough to complete a whole lifecycle on *Bt* corn has been selected so far.

Our second main conclusion is that some predispersal mating does occur for both males and females. Hence, the current HDR management strategy aimed at delaying resistance to transgenic *Bt* corn in ECB populations may not ensure complete mixing between susceptible and resistant moths prior to their first mating. This is all the more important as ECB males are thought to mate on average only 3.8 times over their whole lifetime, producing spermatophores of exponentially decreasing size so that the first copulation accounts for approximately 60% of the total volume emitted over their entire lifetime [ 44].

This lack of complete mixing between susceptible and resistant moths prior to their first mating probably increases the proportion of crossings among homozygous resistant *(rr)* individuals to be expected. Actually, our results point out that the proportion of *rr* × *rr* crossings over the entire landscape strongly depends on the timing and importance of males' long-range dispersal. Indeed, the higher the proportion of *SS* and *rS* males emigrating from the refuges into the pool of males present at the edges of *Bt* cornfields, the lower is the proportion of resident *rr* females that will be mated by *rr* males emerged from the same field. Also, it must be noted that the densities we released mimic a field where the frequency of the resistance allele is already quite high. If the *r* allele is initially as rare as estimated by Bourguet et al. [ 45], it is possible that resistant ECB in *Bt* field borders would be at such low densities that their behavior might be different; they might, for instance, leave more readily than in the present study. On the other hand, it is unlikely that an *rr* individual would be completely alone in a *Bt* field, as some of its sibs should be present, too. Notably, Experiment 1 shows that, within the pool of males present in the field border, the proportion of males that had immigrated from cornfields located several hundred meters away was very variable between sites and between dates: it ranged from 32.8% to 100%. The spatiotemporal variations of the proportion of resident moths staying in their natal field long enough to mate, and of the local proportion of resident to immigrant males in fields with small resident populations, as well as the factors affecting them appear to be crucial points, that must be studied in more detail. Again, it must also be kept in mind that while these factors need to be studied for susceptible moths, results may not be directly applicable to resistant genotypes. For instance, resistant P. gossypiella have been found to display a longer developmental time [ 46], which could affect (positively or negatively) the probability of effective mixing at the landscape level.

The influence of predispersal mating on *Bt* resistance evolution under the HDR strategy is not trivial. While models of the HDR strategy assuming crop rotation conclude that predispersal mating uniformly increases the speed at which *Bt* resistance alleles are selected [ 4], others [ 13, 34, 37, 41] show that, if crops are not rotated, departures from the original assumptions on the life history of the pest (amount of dispersal, timing between dispersal and mating, oviposition, etc.) can also significantly affect the predictions. Notably, they show that the negative consequences of predispersal mating can be offset, provided it is not too high, by non-random oviposition on *Bt* rather than on non- *Bt* crop fields. Provided crops are not rotated (i.e., provided the same fields are used as refuges year after year), such preferential oviposition could result from some females staying in their natal field not only to mate but also to oviposit. However, all these models assume, in addition, sex-biased premating dispersal, with females staying in the vicinity of their natal field until mating and males dispersing and then mating randomly with any of them. Our data provide no evidence that such a sex-bias exists, so that, to be conservative, this assumption warrants more careful checking before it can be made.

Some models assuming no crop rotation suggest that an intermediate amount of dispersal can, in certain cases, be better than low or high dispersal: if the HDR strategy were applied in this framework, a precise quantification of the amount of predispersal mating would become important. Our results do not provide such precise quantification. They offer a method to estimate this proportion, but most of all they show that it can be highly variable, suggesting that it may not be so easy to find a single, generally applicable value and that overestimating or underestimating it with a security margin may not be safely applicable either. Therefore, relying on an intermediate level of predispersal mating in the HDR strategy seems to require more finetuning than can probably be concretely achieved on a large scale. In addition, models using this framework assume that female postmating dispersal is low (i.e., that females staying close to their natal field to mate will also stay there to oviposit), an assumption that may be reasonable but remains yet to be checked. Another method for reducing random ovipositing could be strategic cutting of field borders or other means to bias the attractivity of *Bt* fields versus refuges for oviposition—a “trap-crop” type of strategy already suggested by Alstad and Andow [ 4].

In sum, our results do not necessarily imply that the HDR strategy cannot work. However, they caution that model assumptions must be carefully investigated before relying on them, and they offer a method to do so, which could also be applied to other pests targeted by *Bt* crops.

## Materials and Methods

Statistical analyses were performed using SYSTAT 9.0 software [ 47] or JMP IN 3.0 software [ 48]. Throughout this paper, all *p*-values are given for two-tailed tests. Time is given as local time.

### Experiment 1.

Mark-release-recapture experiments were conducted during the June 2004 breeding season at four sites located in a corn-growing area, approximately 20 km south of Toulouse, France. Adult ECBs were obtained from an outbred strain that was mass-reared on a wheat diet. Male and female pupae were kept separately, so that adults were all virgin at the time of release, which was conducted less than 24 h after emergence. As wheat and corn use different types of photosynthesis (C _3_ and C _4_ type, respectively), the ratio of ^12^C and ^13^C carbon isotopes in their tissues differ [ 28]. This characteristic is transferred to moth tissues [ 27], so that laboratory-reared individuals fed on wheat diet can be distinguished from field individuals fed on corn. In addition to this chemical marking, moths were color-marked prior to release. After approximately 30 min at 6 °C (until they were unable to fly), they were marked on the dorsal thorax and base of the wings with a 1:1 ink/ethanol mixture applied with the tip of a matchstick. A different colour was used for each release and for each of the two age classes released (less than 12 h and 12 to 24 h). After marking, moths were placed into small boxes and stored in cool boxes with ice blocks to reduce agitation, which might cause damage or abnormally high dispersal at the time of release. In each site, the experiment was carried out in a field planted with corn in 2004 and with soybean or wheat in 2003. Because these two crops suffer very little infestation by the ECB [ 26, 27], our experimental set-up mimicked the situation in a cornfield planted with *Bt* corn the year before.

Herbaceous borders along cornfields are described as a very suitable habitat for the ECB, where large numbers of adults typically mate and rest during the daytime [ 49]. During the afternoon before each release, a 100-m section of a herbaceous border running along a cornfield and of the first eight rows into the cornfield was cleared of any moth using sweep net capture. Capture efforts were stopped when the 100-m section could be screened once entirely without finding more than five additional moths. At approximately 08:00 p.m., the small boxes containing the marked moths were taken out of the cool boxes, placed open into a 40-m section along a line parallel to the cleared border section, at equal distance from both ends, and situated between the seventh and eighth row into the cornfield, approximately 7 m from the herbaceous border of the field, and gently agitated until the moths left. Recaptures were conducted by sweep net capture, 36 h later, in the 100-m section cleared before release. Again, all efforts were made to ensure exhaustive capture of all marked and unmarked ECB moths over this section. The proportion of recaptured moths was not influenced by their age (less than 12 h or 12 to 24 h) upon release (paired *t*-test, *t* = 0.002, *df* = 15, *p* = 0.998), so that released moths were pooled regardless of age (less than 24 h) in subsequent analyses.

We dissected all color-marked recaptured females to determine their mating status. We also dissected a subsample ( *n* = 79) of the wild females caught during recapture sessions. In order to estimate their mating rate with marked versus unmarked males conservatively, we took these 79 females from those sessions displaying the highest released/wild males ratios (site 1: 03 June; site 2: 02 June; site 3: 01, 07 and 11 June; site 4: 31 May and 04 June). Finally, for each of four recapture sessions conducted in site 1, we dissected not only the marked but also the unmarked females, and we captured 49 to 55 (unmarked) females in site 5, i.e., in the border of a large cornfield located approximately 400 m away from site 1 that was a likely source of many of the immigrant moths captured there ( Tables S3 and S4). The bursa copulatrix of the genital duct of mated female moths contains one or several spermatophores, i.e., solidified droplets of sperm and nutritious substances deposited by males during the mating process (usually one per mating event [ 44]) and later used by the female. The overall proportion of females mated with wild *vs.* released males was compared with a Fisher exact test between unmarked and marked females. The numbers of spermatophores carried by marked versus unmarked females at site 1 and by unmarked females at site 1 versus site 5 were compared for each replicate by means of a Kruskal-Wallis nonparametric analysis of variance. The shape and color of spermatophores were used to assign them to two age categories (“recent”: white and of ovoid shape, i.e., little digested, versus “old”: brown and with a substantial part missing, indicating a more advanced stage of digestion), from which it can be inferred how recently the mating occurred [ 42, 50]. The proportion of old versus new spermatophores carried by females was compared for each replicate with a Fisher exact test between unmarked and marked females at site 1 and between unmarked females at site 1 and site 5. Global tests across replicates were constructed using Fisher's combined probability test method (Fisher's global test [ 51, 52]) to compare the number of spermatophores and the proportion of old *vs.* new spermatophores carried by the same pairs of groups of females.

Stable carbon isotope analysis of wings, legs, or spermatophores of ECB moths were conducted as described in [ 27]. Results are expressed in conventional “per mill” units relative to the Pee Dee Belemnite standard as: δ ^13^C (‰) = [(R _sample_ − R _standard_)/R _standard_] × 1,000, where R _sample_ and R _standard_ are the ^13^C/ ^12^C atom ratios in the sample and the standard, respectively.

We checked for possible differences in survival and in mating propensity between marked and wild moths in two control experiments: *SURVIVAL* and *MATING*. The *SURVIVAL* experiment was conducted as follows. While we performed Experiment 1, in June 2004, three replicates of five color-marked and five non–color-marked females and five color-marked and five non–color-marked males, all less-than-12-h-old adults from the wheat-fed laboratory strain used in Experiment 1, were placed into a 20 × 20 × 40-cm plastic box with a wet paper wad at room temperature with natural daylight. Deaths were recorded daily. We analysed the number of days of survival using a general linear model with sex, marking, and marking color nested in marking as fixed factors and replicate as a random factor. In laboratory conditions, once the approximately 2-d difference in survival between males and females had been accounted for ( *F*_1,166_ = 43.59, *p* < 0.0001), there was only a slight difference in survival of colour-marked versus unmarked moths, and the difference was in favor of the former (8.32 ± 0.27 d and 7.62 ± 0.26 d, respectively; *F*_1,166_ = 4.67, *p* = 0.032), and all moths survived at least 2 d ( Table S1). Thus, the released moths that were not recaptured in the field were probably mostly still alive.

The *MATING* experiment was conducted as follows. While we performed Experiment 1, in June 2004, four replicates of ten females and ten males, all color-marked and all from the wheat-fed laboratory strain used in Experiment 1, were placed together with ten unmarked wild males collected less than 2 h earlier, next to a cornfield approximately 5 km from the closest study site, into the same boxes and in the same room conditions as replicates of the *SURVIVAL* experiment. We thereby controlled that, in laboratory conditions, (i) when caged with equal proportions of marked and unmarked males, marked (this experiment) and non–color-marked ( *SURVIVAL* experiment) females showed the same overall mating rate after 48 h (χ ^2^ = 3.80, *df* = 3, *p* = 0.284, Table S1), and that (ii) recently emerged (less than 24 h old) marked males had the same probability to mate with marked females as wild males collected directly in the field (Fisher's exact test, *p* = 0.683, Table S2). Thus, released females probably had the same propensity to mate as wild females, and so had released and wild males.

*Data analysis of assortative mating*— we estimated the probability, for a released moth, to mate assortatively with other released moths by assuming that each released virgin female would mate either with a marked, released male or with a wild male present at the study site. Let *p*_A *ij*_ and *f _ij_* be, respectively, the proportion of released females mating assortatively with released males rather than randomly and the proportion of released males among all males present at site *i* during a given release-and-recapture session *j*. As an estimate for *f _ij_*, we used the proportion of marked males among total males captured during the recapture conducted 36 h after the corresponding release). Any female released at site *i* during session *j* has a probability *p*_A *ij*_ + (1 − *p*_A *ij*_) • *f _ij_* of mating with a released male and (1 − *p*_A *ij*_)(1 − *f _ij_*) of mating with a wild male. Using the values of parameters *p*_A *ij*_ and *f _ij_*, it is then possible to compute the likelihood of the observed proportions of marked and unmarked spermatophores retrieved from females captured at each site and for each session. To obtain posterior distributions for *p*_A *ij*_, we applied a Bayesian Markov chain Monte Carlo approach with an uninformative [Uniform (0,1)] prior and lognormal deviates. Ten thousand values were sampled with a thinning of 100 and a burn-in of 10,000 and used to calculate statistics (mode and credibility interval) of the posterior distribution. The 95% credibility interval of *p*_A *ij*_ was estimated as the highest posterior density region, i.e., the region of values that contains 95% of the posterior probability [ 53]. The probability of assortative mating was assumed to be identical for all release sessions and sites.

### Experiment 2.

Experiment 2 was conducted during the June 2005 breeding season, in the same corn-growing area as Experiment 1. Approximately 800 pupae were obtained by mass-rearing the same outbred strain used in Experiment 1. These pupae were placed between the seventh and eighth rows of a cornfield during 11 d (05−15 June), i.e., they were allowed to emerge directly in the field. Experiment 2 proceeded in four independent steps, as follows. Over 5 d of observation, we recorded (i) individual emergence times ( *n* = 221), (ii) the time ( *n* = 169), and (iii) the distance ( *n* = 124) of the first flight performed after emergence. After their first flight, moths were gently placed individually in a small plastic box with a wad of wet paper, which was left in field conditions in the shade. We also recorded (iv) the frequency and distance of subsequent flights during a total of 136 observation sessions of 10 min each, spread from 10:00 a.m. to 09:30 p.m. and over 4 d. During these observation sessions, less-than-24-h-old moths that had already performed their first flight were taken out of the small boxes in which they were kept in field conditions and settled individually on cornstalks. Possible moth movement was monitored for 10 min and the distance covered during each flight (if any) was measured and recorded. For data analyses, plots of residuals versus predicted values were examined and the distribution of residuals was checked for normality. To improve normality of the residuals' distribution, the distances covered during the first flight following emergence were Ln (1 + distance) transformed before testing for any differences related to sex and period of emergence.

### Experiment 3.

Experiment 3 was conducted, during the first (June) and second (August) 2005 breeding seasons, in the same cornfield as Experiment 2. Females released in June 2005 were obtained from the laboratory strain used in Experiment 2. These females were color-marked as described in Experiment 1. Females released in August 2005 were offspring of approximately 80 wild mated females collected in June 2005 in the vicinity of the experimental site. These offspring were not color-marked but were reared on a wheat diet, which provided a biogeochemical marking allowing later checking.

Females to be released were either kept separate from males since pupal stage or checked very frequently and separated just after emergence, which ensured their not being mated prior to release. Less than 24 h after emergence, they were settled individually on every cornstalk or every second stalk of the eighth row running parallel to the nearest field border. The stalks where released moths were settled were clearly identified with plastic labels. Individual boxes in which moths were kept upon release were gently opened, and the tip of a corn leaf was used to invite the moth to climb the stalk and settle on the plant without flying away. When females flew away, we attempted to recapture them immediately and tried again to settle them until they were either successfully settled or lost. A total of 198 and 549 females were settled successfully in June and August respectively, of approximately 220 and 770 attempts. All females were settled just before sunset—i.e., between approximately 9:00 and 9:30 p.m. in June and between approximately 8:00 and 9:00 p.m. in August, a time when their activity in the field, as well as that of wild females in the vicinity, was usually low (AD, SP, and DB, personal observations). ECBs typically start flying at dusk in search of a suitable site to emit pheromones (females) or of a receptive female (males) and settle at various times before dawn, depending notably on temperature. In the present experiment, moths were checked every 30 to 60 min after release, either until dawn (August) or until the temperature dropped to the point that no moth activity was observed anymore in and around the field for more than 1 h, i.e., approximately 4 h after release (June). Any mating pair including a marked female was collected, and its place of collection (either on cornstalks within the release section, or nearby, within approximately 50 m) was recorded. We checked that all females were able to fly the day after the experiment and that they all survived 3 d or longer in the laboratory. In addition, we confirmed their mating status either by checking for the presence of a spermatophore or by recording the laying of fertile eggs.

## Supporting Information

Table S1 Survival of Subsamples of Marked and Unmarked O. nubilalis Moths Used in Experiment 1 Kept in Laboratory Conditions (See Text) and Mating Status of Each Type of Female
Undet., number of females for which the number of spermatophores could not be determined with certainty.
(35 KB DOC)

Table S2 Number of Marked O. nubilalis Females Found to Be Virgin or Mated with Marked (C _3_) or Unmarked (C _4_) Males after Having Been Held in Laboratory Conditions for 48 h in Three Batches of 20 Females, 10 Marked Males, and 10 Unmarked Males Each
Two marking colors (black and green) were tested. One female had mated twice (both times with a C _4_ male), and one female carried a spermatophore for which C _3_/C _4_ status could not be determined. We also checked that wild-caught males displayed an isotope ratio typical for C _4_-fed individuals. On one occasion (of 30), a wild ECB male from a batch collected near a cornfield, approximately 5 km from the closest study site, for a side-experiment (see MATING) was found to have an isotope ratio typical for C _3_-fed individuals. It presumably belonged to a different, morphologically indistinguishable C _3_-feeding ECB taxon. Indeed, Bontemps et al. [ 30] found that more than 90% of wild-caught ECB adults displaying a C _3_ isotope ratio belong to a distinct ECB taxon that is genetically differentiated [ 54], uses a different sex pheromone [ 55], interbreeds very little (less than 5% [ 31, 56]) with individuals feeding on corn, and typically reaches much lower population densities [ 30, 31]. A small number of such individuals may have been present, but they are unlikely to have interfered with our experiment to any extent, given their small densities and low interfertility. Alternatively, an occasional individual belonging to the corn taxon may have developed on a C _3_ plant. However, this does not affect the overall conclusion of our experiment, i.e., there was no evidence for a difference in mating success between wild and laboratory-reared marked males.
(29 KB DOC)

Table S3 Experiment 1 Number of O. nubilalis females found to carry no, one, two, three, or four spermatophores among randomly picked females caught with a net at two different sites and four different dates. Site 1 is a 2004 cornfield planted with wheat in 2003 (see Tables 1 and 2), and site 5 is a 2004 cornfield planted with corn in 2003 and located approximately 400 m from site 1.
(41 KB DOC)

Table S4 Number of “Recent” and “Old” Spermatophores among Spermatophores Dissected from O. nubilalis Females Caught in Two Sites and on Four Dates (see Table S3)
The age of spermatophores indicates how recently the corresponding mating occurred.
(39 KB DOC)

## Abbreviations

*Bt*: Bacillus thuringiensis
ECB: European corn borer ( Ostrinia nubilalis Hübner)
HDR: high-dose refuge
