# Supplementary material for: Resistance Evolution to Bt Crops: Predispersal Mating of European Corn Borers
Source: PLoS Biol. 2006 May 30;4(6):e181. doi: 10.1371/journal.pbio.0040181 (PMC1470457; doi:10.1371/journal.pbio.0040181)
Supplement: Supplementary file 1 [file pbio.0040181.st001.doc]

**Table S1.** Survival of subsamples of marked and unmarked *Ostrinia nubilalis* moths used in Experiment 1 kept in laboratory conditions (see text) and mating status of each type of female. Undet. = number of females for which the number of spermatophores could not be determined with certainty

| Marking status | Survival (days) | | | | | | |  | Mating status  (number of spermatophores) | | | | |
| --- | --- | --- | --- | --- | --- | --- | --- | --- | --- | --- | --- | --- | --- |
|  | Males | | |  | Females | | |  |  |  |  |  |  |
|  | *n* | mean | SE |  | *n* | mean | SE |  | undet. | 0 | 1 | 2 | 3 |
| Unmarked | 45 | 8.33 | 2.45 |  | 45 | 6.91 | 1.31 |  | 3 | 7 | 21 | 11 | 3 |
| Marked | 45 | 9.73 | 2.71 |  | 45 | 6.91 | 1.99 |  | 2 | 5 | 30 | 7 | 1 |
