# Supplementary material for: Resistance Evolution to Bt Crops: Predispersal Mating of European Corn Borers
Source: PLoS Biol. 2006 May 30;4(6):e181. doi: 10.1371/journal.pbio.0040181 (PMC1470457; doi:10.1371/journal.pbio.0040181)
Supplement: Supplementary file 2 [file pbio.0040181.st002.doc]

**Table S2.** Number of marked *Ostrinia nubilalis* females found to be virgin, or mated with marked (C_3_) or unmarked (C_4_) males after having been held in laboratory conditions for 48 hours in three batches of 20 females, 10 marked males and 10 unmarked males each. Two marking colors (Black and Green) were tested. One female had mated twice (both times with a C_4_ male), and 1 female carried a spermatophore for which C_3_/C_4_ status could not be determined. We also checked that wild-caught males displayed an isotope ratio typical for C_4_-fed individuals. On one occasion (out of 30), a wild ECB male from a batch collected near a cornfield, *ca*. 5km away from the closest study site, for a side-experiment (see *MATING*) was found to have an isotope ratio typical for C_3_-fed individuals. It presumably belonged to a different, morphologically indistinguishable C_3_-feeding ECB taxon. Indeed, Bontemps et al. [30] found that *>* 90% wild-caught ECB adults displaying a C_3_ isotope ratio belong to a distinct ECB taxon that is genetically differentiated [54], uses a different sex pheromone [55], interbreeds very little (< 5% [31,56]) with individuals feeding on corn, and typically reaches much lower population densities [30,31]. A small number of such individuals may have been present but they are unlikely to have interfered with our experiment to any extent, given their small densities and low interfertility. Alternatively, an occasional individual belonging to the corn taxon may have developed on a C_3_ plant. However, this does not affect the overall conclusion of our experiment, *i.e.*, that there was no evidence for a difference in mating success between wild and laboratory-reared marked males

| Color | *n* | *n* virgin | *n* mated by a C_3_ male | *n* mated by a C_4_ male |
| --- | --- | --- | --- | --- |
| Black | 20 | 6 | 6 | 7 |
| Black | 20 | 7 | 7 | 6 |
| Green | 20 | 6 | 5 | 9 |
| All | 60 | 19 | 18 | 22 |
