# Supplementary material for: Resistance Evolution to Bt Crops: Predispersal Mating of European Corn Borers
Source: PLoS Biol. 2006 May 30;4(6):e181. doi: 10.1371/journal.pbio.0040181 (PMC1470457; doi:10.1371/journal.pbio.0040181)
Supplement: Supplementary file 3 [file pbio.0040181.st003.doc]

**Table S3. Experiment 1. Number of *Ostrinia nubilalis* females found to carry 0, 1, 2, 3 or 4 spermatophores among randomly picked females caught with a net at two different sites and four different dates. Site 1 is a 2004 cornfield planted with wheat in 2003 (see Tables 1 and 2), and Site 5 is a 2004 cornfield planted with corn in 2003 and located *ca*. 400m away from Site 1**

|  | Site 5 wild females | | | | |  | Site 1 wild females | | |  | Site 1 marked females | | |
| --- | --- | --- | --- | --- | --- | --- | --- | --- | --- | --- | --- | --- | --- |
| Date | 0 | 1 | 2 | 3 | 4 |  | 0 | 1 | 2 |  | 0 | 1 | 2 |
| 03 June | 0 | 48 | 1 | 0 | 0 |  | 1 | 26 | 3 |  | 2 | 45 | 0 |
| 09 June | 4 | 44 | 2 | 0 | 0 |  | 0 | 26 | 4 |  | 0 | 19 | 0 |
| 13 June | 0 | 45 | 6 | 0 | 0 |  | 0 | 35 | 15 |  | 0 | 11 | 0 |
| 18 June | 1 | 43 | 9 | 1 | 1 |  | 0 | 39 | 10 |  | 0 | 7 | 0 |
