# Supplementary material for: Resistance Evolution to Bt Crops: Predispersal Mating of European Corn Borers
Source: PLoS Biol. 2006 May 30;4(6):e181. doi: 10.1371/journal.pbio.0040181 (PMC1470457; doi:10.1371/journal.pbio.0040181)
Supplement: Supplementary file 4 [file pbio.0040181.st004.doc]

**Table S4.** Number of “recent” and “old” spermatophores among spermatophores dissected from *Ostrinia nubilalis* females caught in two sites and on four dates (see Table S3). The age of spermatophores indicates how recently the corresponding mating occurred

|  | Site 5 wild females | |  | Site 1 wild females | |  | Site 1 marked females | |
| --- | --- | --- | --- | --- | --- | --- | --- | --- |
| Date | recent | old |  | recent | old |  | recent | old |
| 03 June | 45 | 5 |  | 9 | 23 |  | 47 | 0 |
| 09 June | 40 | 9 |  | 13 | 21 |  | 19 | 0 |
| 13 June | 55 | 2 |  | 52 | 12 |  | 9 | 2 |
| 18 June | 53 | 11 |  | 36 | 19 |  | 7 | 0 |
